# You Can Do That? Performing Total Recanalization of Completely Stented Arterial-venous Grafts

**DOI:** 10.7759/cureus.3396

**Published:** 2018-10-01

**Authors:** Taylor S Harmon, Travis E Meyer, Jerry Matteo

**Affiliations:** 1 Interventional Radiology, The University of Texas Medical Branch, Galveston, USA; 2 Interventional Radiology, University of Florida College of Medicine, Jacksonville, USA

**Keywords:** thrombolysis, covered stents, thrombosis, hemodialysis, angioplasty, interventional radiology, percutaneous, venous access, pseudoaneurysm, av graft

## Abstract

Establishing venous access in chronic dialysis patients is conducted by the insertion of polytetrafluoroethylene arterial-venous (AV) grafts. The continual access of these grafts allows for potential failure over extended periods of hemodialysis treatment, as a result of thrombosis and pseudoaneurysm formation. Patency of AV grafts requires interventional management of thrombosis and pseudoaneurysm formation, including thrombectomy and covered stent placement, respectively. In 2013, the Food and Drug Administration approved the Gore REVISE study, giving indication for covered stents within AV grafts. If covered stent placement is required to treat a thrombosed AV graft, it is still possible to perform a percutaneous thrombectomy procedure afterwards. Direct access of the AV graft by passing through both the graft material and covered stents allows for interventional radiology management to be performed without compromise of the stent or graft. This interventional method of direct access can salvage the AV graft before considering further invasive management, such as a new surgical venous access site.

## Introduction

Maintaining venous access in chronic hemodialysis (HD) patients can be very challenging. Various methods such as trans-lumbar inferior vena cava access, dual-lumen central venous catheter placement, and extremity surgical graft placement have all been implemented to acquire adequate venous access in HD patients. In the case of arterial-venous (AV) grafts, complications such as thrombosis within the graft are well-documented in the literature [1]. An interventional percutaneous thrombectomy involves directly accessing the graft lumen by puncturing the graft material in an antegrade and retrograde fashion. Subsequently, mechanical thrombectomy, angioplasty, embolectomy and covered stent placement can be performed. This form of interventional management is an appropriate approach for treating thrombosed HD grafts [2].

Once a current AV graft has become significantly thrombosed and dysfunctional, it is readily acceptable to attempt covered stenting or thrombolysis to maintain graft patency [3]. We propose another method in addition to these under the circumstance that an AV graft has been completely repaired with multiple covered stents. This option warrants necessity when attempting to save a thrombosed stent-lined AV graft in chronically dialyzed patients.

## Technical report

**Figure 1:**
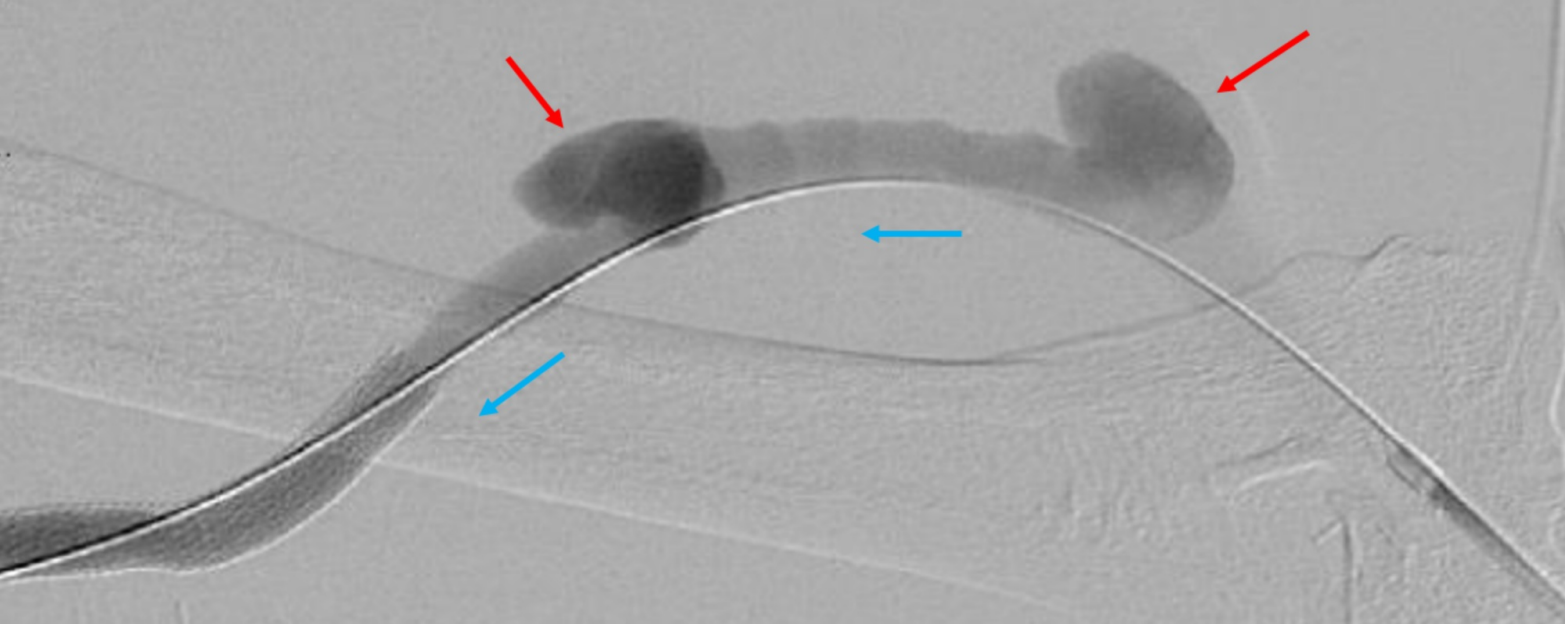
Fistulogram of Arterial-Venous Graft After Six Months. Fistulogram of the six-month-old arterial-venous graft reveals two pseudoaneurysms (red arrow). The blood flow through the graft is in the direction of the blue arrows.

For the aforementioned percutaneous declot of an AV graft that is covered stent lined, a 51-year-old African American male with a past medical history of hypertension and end stage renal disease (ESRD) requiring permanent dialysis is presented. For hemodialysis access, the patient required a polytetrafluoroethylene AV graft that was placed in his left upper extremity. The patient received dialysis through the AV graft without incident until six months later, when the graft was found to have pseudoaneurysms (Figure 1).

**Figure 2:**
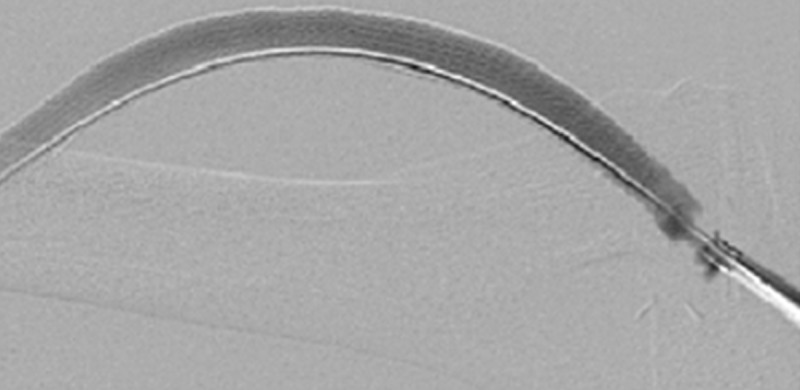
Fistulogram Arterial-Venous Graft With Covered Stents. The graft is seen here completely lined by three covered stents, having excluded the prior seen pseudoaneurysms.

The graft wall was accessed with a micropuncture kit. At this time, it was determined that two Viabahn (W.L. Gore & Associates, Flagstaff, AZ) covered stents were necessary to exclude the pseudoaneurysms (Figure 2).

**Figure 3:**
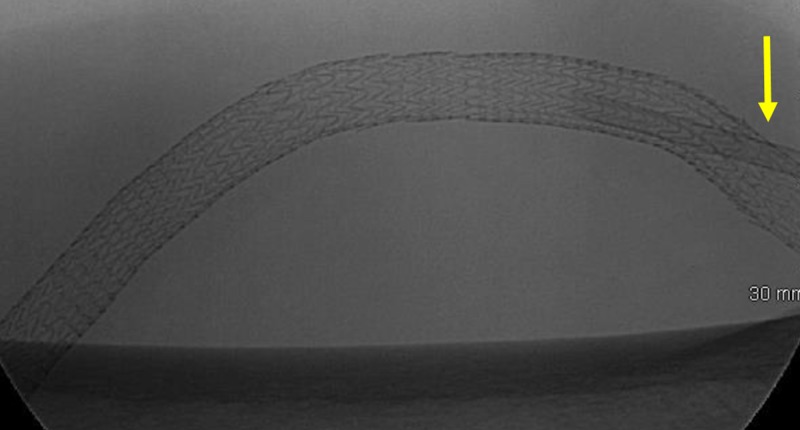
Direct Percutaneous Access of a Multi-Covered Stent Arterial-Venous Graft. The sheath is seen directly accessing both the venous limb and covered stent of the arterial-venous graft (yellow arrow).

Six months later, the patient returned and reported AV graft malfunction. Due to the patient’s medical history, age, and graft condition, it was decided to attempt direct percutaneous access of the graft, though it was lined with multiple covered stents instead of abandoning the graft (Figure 3).

**Figure 4:**
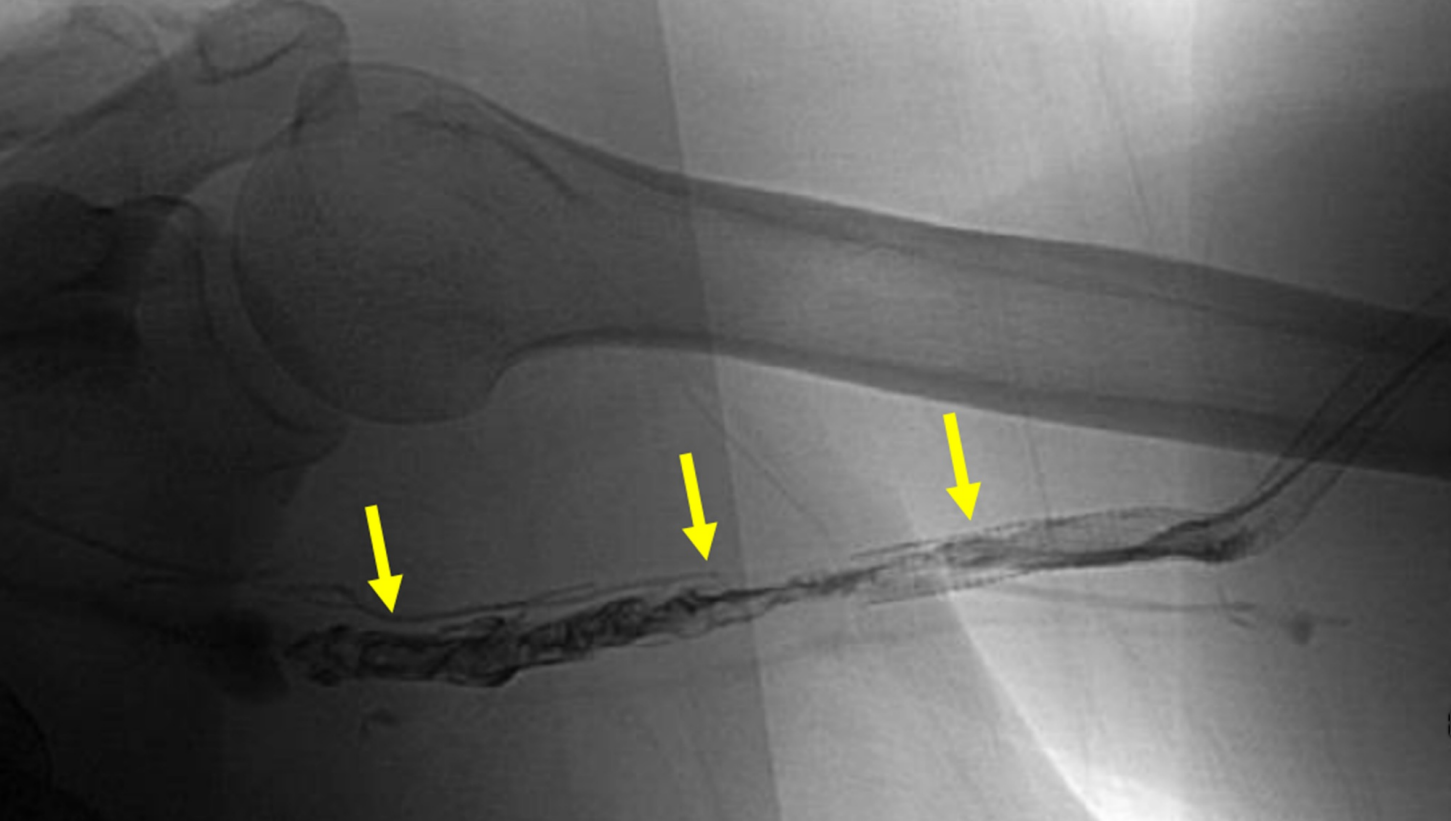
Significant Venous Outflow Thrombosis. The yellow arrows show thrombosis throughout the venous outflow.

Initially using a cross-catheter technique for the declot procedure, the patient’s AV graft was accessed with 19-gauge needles at the proximal and distal aspects of the venous limb, through the existing covered stents aligning the venous outflow. Hand injection venogram at the distal venous outflow revealed thrombus formation (Figure 4).

**Figure 5:**
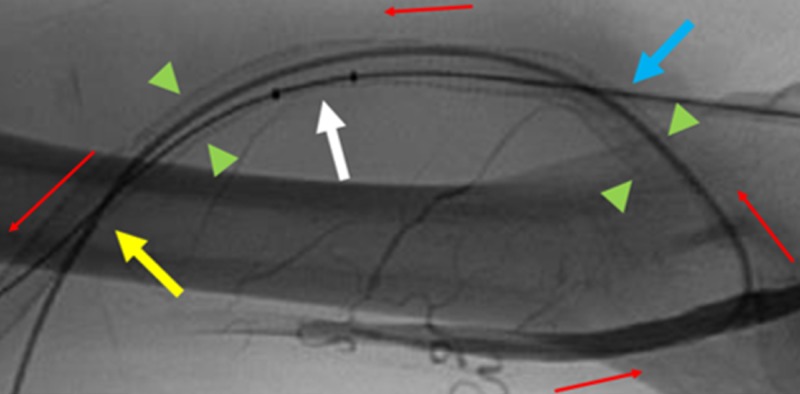
Anatomically Labeled Native Arterial-Venous Graft. The red arrows represent graft blood flow from arterial to venous outflow. The green arrowheads represent the span of the graft that is lined with covered stents starting from the arterial limb to the distal venous outflow. The direct arterial and venous access sites are labeled by the yellow and blue arrows, respectively. An AngioJet thrombectomy device is shown within the graft depicted by the white arrow.

A Bentson guidewire was advanced antegrade across the venous anastomosis site, and a seven French sheath was placed. An angled tip Glidewire (Terumo, Somerset, NJ) was advanced retrograde across the arterial anastomosis site, and a Fogarty balloon catheter was advanced over the wire into the distal brachial artery. Pre-angioplasty of the venous anastomotic site was performed using a 6 mm by 40 mm balloon. The angioplasty catheter was then exchanged for an AngioJet thrombectomy catheter (Boston Scientific, Marlborough, MA), which was used to remove the extensive thrombosis from the venous limb of the graft. The Fogarty balloon catheter was then deployed at the arterial anastomosis, and used to retrieve clot from the arterial limb into the venous limb for AngioJet removal (Figure 5).

**Figure 6:**
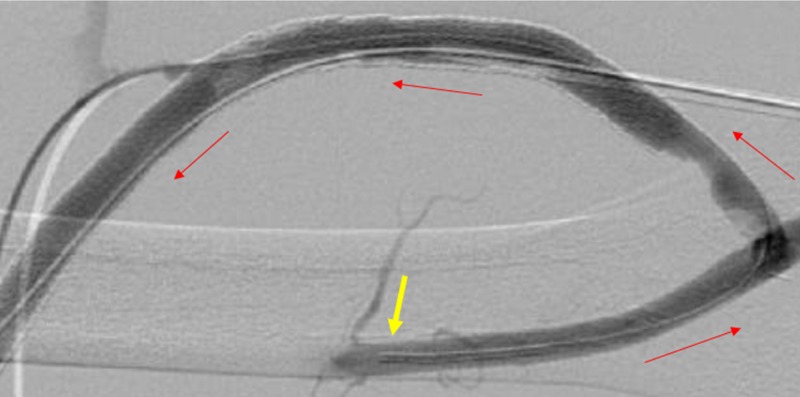
Post-Interventional Fistulogram. A fistulogram is shown of the declotted patent arterial-venous graft, with the direction of blood flow shown by the red arrows. A catheter is shown in the native artery from the venous limb access site in a retrograde fashion (yellow arrow).

The arterial anastomosis also demonstrated high-grade stenosis, decreasing blood flow and prompting angioplasty (Figure 6).

**Figure 7:**
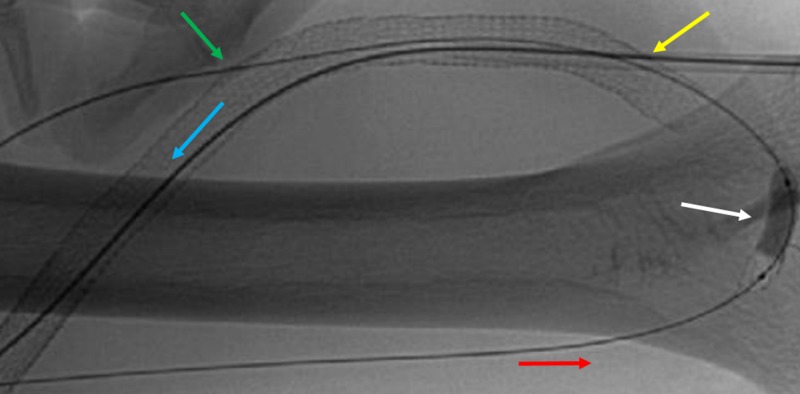
Arterial-Venous Graft Angioplasty. The red arrow represents the blood flow through the graft in the distal brachial artery. The blue arrow represents blood flow through the graft in the venous limb. The arterial and venous direct graft access points are shown as represented by the green and yellow arrows, respectively. An angioplasty balloon is seen at the arterial anastomosis site (white arrow).

A 5 mm cutting balloon was utilized within the anastomosis (Figure 7).

**Figure 8:**
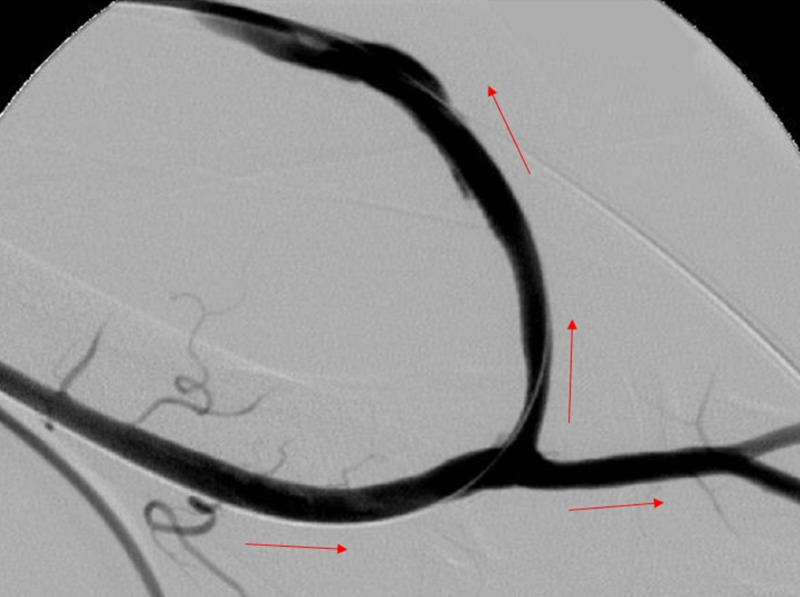
Final Fistulogram Following Declot. A final fistulogram is shown of the arterial-venous graft with patency of the arterial anastomosis and arterial limb. The blood flows through the graft and into the venous limb (red arrows).

Follow-up angiogram from the brachial artery to evaluate the arterial anastomosis showed excellent results with no residual stenosis (Figure 8).

## Discussion

Thrombosis of AV grafts is the most frequently encountered complication for patients receiving chronic hemodialysis, occurring in one-fourth to two-thirds of this patient population [4]. AV grafts remain patent for an average of 10 months before significant thrombosis occurs, and either surgical or interventional management must be considered [5]. For those with ESRD, HD access is the leading cause for hospitalization [6]. For these reasons as well as the increasing number of patients on chronic dialysis, it is important to continue exploring new interventional methods for preservation of AV grafts.

Historically, patients have managed to salvage the function of their stented AV grafts by receiving various methods of declot intervention, including the majority of our patients at our institution [7]. Following thrombectomy, AV grafts have been shown to remain patent for three months in 55.9% of patients following initial intervention, in 61.9% of patients following a second intervention, and in 55.8% of patients following a third intervention [8]. This data suggests that percutaneous management of significantly thrombosed AV grafts should be implemented, in order to prolong the function of these grafts for as long as possible [8]. Furthermore, this infers that attempting interventional declot by percutaneously accessing the AV graft directly is a viable option to perform. After all, these covered stent-lined grafts are continually accessed multiple times a week for HD. The physical repeated access of AV grafts for HD has not shown to cause complications in function [9].

The alternative to the proposed percutaneous declot procedure of covered stents in existing AV grafts is to access a new HD site by catheter placement or de novo creation. It is not uncommon that access options are limited despite the necessity for hemodialysis once a native AV has been significantly or completely thrombosed. Patients will often receive imperative but risky procedures that include surgical establishment of new AV grafts, in order to acquire the venous access that is needed for further hemodialysis [10]. Additionally, these surgical procedures may require burdensome hospital stays, where the increased risk for infection in these patients who are already immunocompromised, increases [11]. Understanding the indispensable value for maintaining a patent site for HD access is paramount in chronically dialyzed patients. The proposed method for percutaneous interventional management of covered stented AV graft maintenance, applied under the appropriate circumstances, is a novel and advantageously therapeutic intervention. As presented, this interventional method is shown to be less invasive over the traditional surgical establishment of new venous access sites in patients requiring continual hemodialysis.

Possible candidates for the proposed intervention presented here include any patient with a graft lined by covered stents. It has been explained that primary and secondary declotting of grafts is preferred over the creation of a new venous access site. Therefore, it is suggested that the direct percutaneous access of a covered stent-lined graft for thrombectomy should be considered. In an extensive literature search, specific examples of the intervention presented here could not be found, but it is suspected that interventions as such have been attempted successfully.

## Conclusions

The most common complication in chronically hemodialyzed patients is AV graft malfunction, as a result of significant or complete thrombosis. With the recent Food and Drug Administration approval of the Gore REVISE clinical study for covered stents within AV grafts, the number of reinterventions will increase. With this approval, the described techniques presented in this article will be routine in the future. Traditional methods to maintain hemodialysis access include risky and invasive management, such as surgical establishment of new venous access sites. Surgical intervention as such leads to prolonged hospital stays, increased risk for infection, and other unnecessary or inconvenient complications. To eliminate the risk of superfluous management in chronic hemodialysis patients, interventional percutaneous thrombectomy of multiple-lined cover stented AV grafts should be performed in order to salvage the function of the graft. Furthermore, this may prevent the necessity for establishing new venous access sites, and avoiding additional invasive surgical procedures altogether.
